# Electricity in the Treatment of Epilepsy

**Published:** 1887-06

**Authors:** 


### ELECTRICITY IN THE TREATMENT OF EPILEPSY.

Dr. A. D. Rockwell, of New York, recently read a very interesting paper before the Academy of Medicine of that city on this interesting subject. The following were the conclusions at which he arrived:

1. Electricity possesses a certain value in the treatment of epilepsy. It is not known, nor is it alleged, that used alone it can cure epilepsy. Used in connection with the bromides, however, its value is unmistakable, and under its use a certain proportion of patients will recover that otherwise would fail to do so.

2. It is in the nocturnal variety of epilepsy more especially that the good effects of electricity are seen, although day attacks have been successfully controlled.

3. The methods of electrical treatment that have proved most efficacious in my hands are central galvanization and general faradization.

4. When electricity fails to cure, or aid in the cure, it is often efficacious, by the method of general faradization, in affording grateful relief from nervous symptoms of an indefinable subjective character; in other words, from that general instability of the nervous system recognized under the term neurasthenia.

5. The systematic use of electricity renders the system more tolerant of the bromides, and will diminish bromic acue.

6. It is important that electrical treatment should be administered with care and judgment; especially should all interruptions of the current be avoided in central galvanization, as the resultant shock is liable to hasten rather than prevent an attack.

7. Two years must elapse without an attack before any case of epilepsy can be considered as one of positive cure.—*College and Clinical Record.*

